# Spontaneous necroptosis and autoinflammation are blocked by an inhibitory phosphorylation on MLKL during neonatal development

**DOI:** 10.1038/s41422-021-00583-w

**Published:** 2021-11-02

**Authors:** Xinxin Zhu, Na Yang, Yu Yang, Feiyang Yuan, Dandan Yu, Yu Zhang, Zhaoqian Shu, Ning Nan, Hong Hu, Xiaoyan Liu, She Chen, Liming Sun, Huayi Wang

**Affiliations:** 1 School of Life Science and Technology, ShanghaiTech University, Shanghai, China; 2 State Key Laboratory of Cell Biology, CAS Center for Excellence in Molecular Cell Science, Shanghai Institute of Biochemistry and Cell Biology, Chinese Academy of Sciences, University of Chinese Academy of Sciences, Shanghai, China; 3 University of Chinese Academy of Sciences, Beijing, China; 4 National Institute of Biological Sciences, Beijing, China; 5 Tsinghua Institute of Multidisciplinary Biomedical Research, Tsinghua University, Beijing, China

**Keywords:** Necroptosis, Developmental biology

Dear Editor,

Necroptosis, a form of caspase-independent cell death, is tightly regulated to maintain tissue homeostasis. The execution of necroptosis depends on receptor interacting protein kinase 3 (RIP3)-activated mixed lineage kinase domain-like protein (MLKL). MLKL is phosphorylated by RIP3, which releases MLKL autoinhibition and drives its self-oligomerization.^1–3^ Oligomerized MLKL translocates to cellular membranes, where it disrupts membrane integrity causing necroptotic cell death.^2,3^

The activation of MLKL is heavily controlled by phosphorylation-driven regulations. Besides the RIP3-dependent MLKL phosphorylation in cytosol, the membrane-associated TAM kinase was reported to phosphorylate MLKL and promote its further oligomerization on membrane, facilitating necroptosis execution.^4^ Previous mutagenesis studies implied the existence of potential inhibitory phosphorylation on MLKL.^5^ However, the physiological relevance of inhibitory phosphorylation on MLKL is largely unknown. Taking into account the commonly observed phenomenon that in vitro necroptosis induction cannot inflict 100% cell death in any cell lines, we harvested the necroptosis surviving cells, which were assumed to enrich the potential negative regulatory signals, and performed SILAC experiment to quantitatively characterize the phosphorylation modifications on MLKL (as illustrated in Fig. 1a). Mass spectrometric analysis identified a single phosphorylated MLKL peptide FSNRpSNICR with Ser83 phosphorylation (Fig. 1b). We then introduced single site mutation to test how it affects TNF-induced cell death. It turned out that the TNF-induced necroptosis, not apoptosis, was specifically blocked by the phospho-mimic mutation of MLKL (S83D or S83E) (Supplementary information, Fig. S1a). Overexpressing MLKL-S83E in the wild-type (WT) MLKL-expressing cells could also obstruct necroptosis (Supplementary information, Fig. S1b, c). In contrast, phospho-dead mutant MLKL-S83G could reconstitute necroptosis as WT MLKL in the *MLKL*-knockout HT29 cells (Fig. 1c). Albeit with low expression levels, the S83N, S83R, S83K and S83C MLKL mutants still partially restored WT MLKL’s function; in contrast, S83Q, S83V, S83L, S83I and S83A mutants failed to reconstitute necroptosis (Fig. 1c). Notably, the site of human MLKL-Ser83 is conserved across species, such as chimpanzee, mouse, rat, hamster, bovine, and pigeon (Fig. 1d). We then introduced mouse MLKL-Ser82 (S82D and S82E) mutations into *Mlkl*-knockout NIH/3T3-RIP3 cells. Consistently, necroptosis was also greatly inhibited in this mouse cell line (Supplementary information, Fig. S1d). Thus, this necroptosis-inhibitory role of the phosphorylation on hMLKL-S83 or mMLKL-S82 is conserved in human and mouse.

**Fig. 1:**
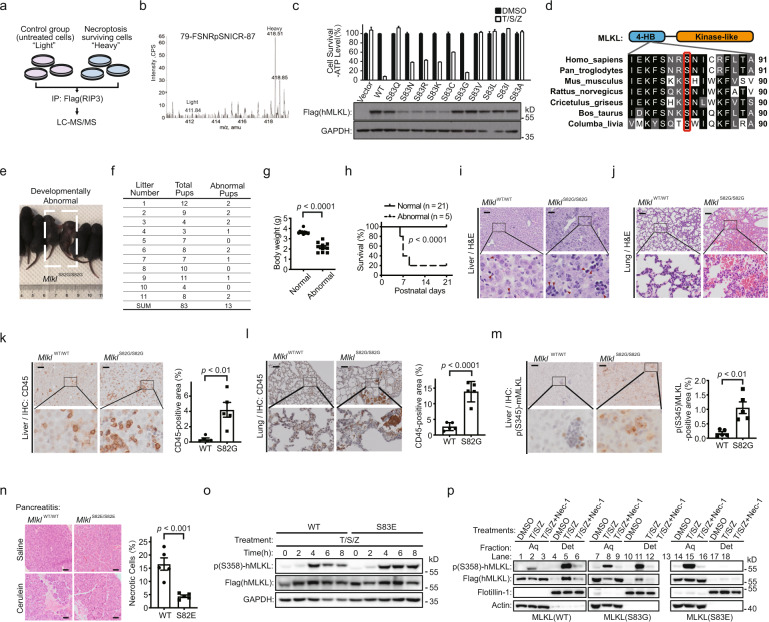
Phosphorylation on MLKL-Ser82 blocks spontaneous necroptosis and autoinflammation during neonatal development via inhibiting MLKL activity at downstream of RIP3-mediated MLKL activation. **a** SILAC workflow. RIP3-expressing HeLa cells (HeLa-RIP3) were grown in SILAC DMEM. Necroptosis was induced by administrating cells with T/S/Z (T, TNF; S, Smac mimetic; Z, z-VAD) for 16 h. SILAC experiment was performed as described in detail in Supplementary information, Data S1. **b** Quantification of MLKL phosphorylation peptide from HeLa-RIP3 cells. A light and heavy isotope-labeled peptide pair was matched to a MLKL single phosphorylated peptide, and the heavy/light peptide ratio was larger than 10 based on the XIC of this peptide pair. **c** Point mutation screening for the Ser83 phospho-dead mutant of human MLKL. *MLKL*-knockout HT29 cells were infected with lentiviral vectors encoding Flag-tagged WT or Ser83-mutant MLKL as indicated. Necroptosis was induced by treating cells with T/S/Z for 16 h. Cell viability was evaluated by measuring intracellular ATP levels using CellTiter-Glo assay (upper panel). The data are represented as means ± SD of duplicate wells. The expression of MLKL mutants was analyzed by immunoblotting (lower panel). **d** Sequence alignment of conserved serine in the MLKL orthologs across different species. The conserved sequence flanking the human MLKL-S83 was used as query sequence in a gapped BLAST search. The invariant serine residues were squared in red. **e** Representative image of a litter of S82G mutant (*Mlkl*^S82G/S82G^) mice at postnatal day 9 (P9). The developmentally abnormal S82G mutant mice were labeled with a dashed, showing smaller size and physical features. **f** Distribution of developmentally abnormal offsprings from *Mlkl*^S82G/S82G^ homozygous intercrosses. **g** Body weight of *Mlkl*^S82G/S82G^ mice at postnatal day 8 (P8). Each dot represents an individual mouse (*n* = 10 per group). Data are means ± SEM. *P* values were determined by two-tailed unpaired *t*-test. **h** Kaplan-Meier survival curve of developmentally normal and abnormal *Mlkl*^S82G/S82G^ mice. *P* values were determined by two-sided log-rank test. **i**, **j** Representative images of H&E staining of liver (**i**) and lung (**j**) sections from *Mlkl*^WT/WT^ and developmentally abnormal *Mlkl*^S82G/S82G^ mice at P8. Arrows, infiltrated immune cells. Scale bar, 50 µm. **k**, **l** Representative images of immunohistochemistry (IHC) staining with anti-CD45 antibody on liver (**k**) and lung (**l**) sections from *Mlkl*^WT/WT^ and developmentally abnormal *Mlkl*^S82G/S82G^ mice at P8. Scale bar, 50 µm. Three different views were counted for each mouse. Corresponding quantifications of infiltrated CD45^+^ signals are shown on the right. Each dot represents the data from an individual mouse (*n* = 5 per group). Data are means ± SEM. *P* values were determined by two-tailed unpaired *t*-test. **m** Representative images of IHC staining with anti-phospho-mMLKL-S345 antibody on liver sections from *Mlkl*^WT/WT^ and developmentally abnormal *Mlkl*^S82G/S82G^ mice at P8. Scale bar, 50 µm. Three different views were counted for each mouse. Corresponding quantifications of the activated MLKL (labeled with phospho-mMLKL-S345 antibody) signals are shown on the right. Each dot represents the data from an individual mouse (*n* = 5 per group). Data are means ± SEM. *P* values were determined by two-tailed unpaired *t*-test. **n** Phospho-mimic mutation of mMLKL-Ser82 plays a protective role in cerulein-induced acute pancreatitis. Scale bar, 50 µm. Five different views were counted for each mouse. Corresponding quantifications of the necrotic cells are shown on the right. Each dot represents the data from an individual mouse (*n* = 5 per group). Data are means ± SEM. *P* values were determined by two-tailed unpaired *t*-test. **o** Immunoblotting analysis of RIP3-mediated MLKL activation using whole cell lysates from necroptotic WT and phospho-mimic (S83E) MLKL-expressing HeLa-RIP3 cells. Necroptosis was induced by administrating cells with T/S/Z for the indicated times as shown in Supplementary information, Fig. S3a. The specific RIP3-mediated MLKL phosphorylation was analyzed by immunoblotting with the anti-phospho-hMLKL-T357/S358 antibody. **p** Triton X-114 phase fractionation analysis of WT and phospho-S83 mutant MLKL subcellular distribution upon necroptosis induction. β-actin and caveolar membrane protein flotillin-1 served as loading controls for cytosolic protein and membrane-integrated protein, respectively.

To investigate the in vivo function of this necroptosis-inhibitory phosphorylation on MLKL, we generated the knock-in mice with phospho-dead S82G mutation of mMLKL (Supplementary information, Fig. S2a). Interestingly, 16% *Mlkl*^S82G/S82G^ offspring appeared normal at birth but became runted as early as five days after birth and died before weaning (Fig. 1e–h; Supplementary information, Fig. S2b). These abnormal *Mlkl*^S82G/S82G^ mice all developed severe inflammation in multiple organs, such as liver and lung (Fig. 1i, j), featuring an increase of infiltrated CD45^+^ inflammatory cells (Fig. 1k, l). It is worth noting that the lungs of developmentally abnormal *Mlkl*^S82G/S82G^ mice exhibited severe pathological damage, including diffuse alveolar damage, thrombotic vasculopathy, and pulmonary hemorrhage (Fig. 1j). These mice also displayed dyspnea, suggesting that fatal respiratory failure led to their neonatal death. In line with these inflammatory phenotypes, immense activated RIP3 (pS232-RIP3) and RIP3-mediated phosphorylation on MLKL-S345 (pS345-MLKL) signals were detected in the inflammatory organs of the developmentally abnormal *Mlkl*^S82G/S82G^ mice (Supplementary information, Fig. S2c; Fig. 1m). mRNA of TNF, one of the necroptosis-inducing factors, elevated obviously, suggesting that it underwent auto-amplification loop of necroptosis and inflammation (Supplementary information, Fig. S2d).^6^ Some of pS345-MLKL signals specifically clustered as puncta on the plasma membrane (Fig. 1m; Supplementary information, Fig. S2e), indicating MLKL activation and necroptosis occurrence. In sum, these data indicated that phosphorylation on mMLKL-Ser82 is required for normal development of mice by preventing necroptotic inflammatory injuries and neonatal death.

In contrast to the developmentally abnormal population of *Mlkl*^S82G/S82G^ mice, there is a group of *Mlkl*^S82G/S82G^ offspring that were born with normal weight and matured well (Fig. 1e, f). By immunoblotting and qRT-PCR analyses, these developmentally normal *Mlkl*^S82G/S82G^ mice were found to have significantly decreased or even undetectable protein and mRNA transcript levels of MLKL in multiple organs compared to WT mice (Supplementary information, Fig. S2f, g). Importantly, although these MLKL-decreased *Mlkl*^S82G/S82G^ mice survived to adults, they showed varying degrees of liver inflammatory injury at the postnatal early stage (Supplementary information, Fig. S2h), which was phenotypically consistent with those neonatally dead *Mlkl*^S82G/S82G^ mice. As long as these mice matured to adults and their MLKL expression level became undetectable, the inflammatory injuries were attenuated concurrently. These data indicate that a negative feedback loop exists to downregulate MLKL expression when MLKL is aberrantly activated during neonatal development.

Using the same strategy as generation of S82G mice, we generated the phospho-mimic mutant *Mlkl* knock-in mice (*Mlkl*^S82E/S82E^) (Supplementary information, Fig. S2a). The *Mlkl*^S82E/S82E^ mice underwent normal development (Supplementary information, Fig. S2i), and the number of weaned mice with S82E mutation strictly conformed to Mendelian ratios (Supplementary information, Fig. S2j). The expression level of MLKL in *Mlkl*^S82E/S82E^ mice was comparable to that in WT mice (Supplementary information, Fig. S2k), and the MDFs and BMDM cells derived from these mice were protected from TNF-induced necroptosis (Supplementary information, Fig. S2l, m). It was reported that *Mlkl*-knockout mice could be protected from cerulein-induced acute pancreatitis.^7^ Similar to the *Mlkl*-knockout mice, we found that pancreas acinar cell loss and inflammation injury were largely inhibited in *Mlkl*^S82E/S82E^ mice after cerulein injection (Fig. 1n). In sum, the phosphorylation of mMLKL-Ser82 protected mice from MLKL-mediated developmental and pathological injuries.

The Ser83 located in the N-terminal helix bundle domain of MLKL (Fig. 1d). Previous studies reported that the double mutation of mouse MLKL-K80AK81A or covalent targeting of human MLKL-C86 by Necrosulfonamide (NSA) all blocked necroptosis, suggesting the importance of this region for MLKL function.^1,8^ Consistent with the observation that NSA binding blocked necroptosis but did not affect the regulations on the C-terminus kinase-like domain, such as RIP3-mediated phosphorylation (pT357/S358),^1^ we also found that the cell death was blocked but RIP3-mediated MLKL phosphorylation was not affected by the S83E mutation over time (Supplementary information, Fig. S3a; Fig. 1o). In line with this, RIP3–MLKL complex formation was not affected by either phospho-mimic or phospho-dead mutations on S82/83 upon necroptosis induction by TNF (Supplementary information, Fig. S3b, c). Moreover, in a RIP3-free system, phospho-mimic mutant MLKL (S82/83E) blocked cell death induced by AP20187-driven MLKL dimerization (Supplementary information, Fig. S3d, e). The compound mutant MLKL-S345E/S82E showed impaired necroptosis activities compared to WT or S345E mutant MLKL (Supplementary information, Fig. S3f). These data indicated that phosphorylation on human MLKL-Ser83 or mouse MLKL-Ser82 inhibits MLKL activity at downstream of RIP3-mediated MLKL activation.

Necroptosis execution depends on membrane integration of RIP3-activated MLKL. Using Triton-114 phase separation assay, we found that the phospho-mimic mutant MLKL-S83E could not integrate into the detergent phase as WT or S83G mutant MLKL (Fig. 1p; Supplementary information, Fig. S3g). The direct consequence of MLKL membrane integration is membrane damage. Using the liposome leakage assay, we found that the membrane-damaging ability of recombinant proteins of phospho-mimic mutant MLKL-S83E was largely decreased compared to WT or S83G mutant MLKL proteins (Supplementary information, Fig. S3h). Thus, our study revealed a novel necroptosis checkpoint that specifically restricts MLKL membrane integration to prevent the occurrence of spontaneous necroptosis during neonatal development. Interestingly, homozygous mice harboring a single mutation of mMLKL-D139V were also reported to develop lethal neonatal inflammation.^9^ It is worth noting that the D139V mutation acts as a dominant active mutation for MLKL, while the S82/83 G mutation is not (Fig. 1c). Interestingly, the expression level of MLKL-D139V was posttranslationally decreased in the homozygous mice, suggesting that there might exist shared downregulation of MLKL expression at transcriptional or post-translational level when MLKL is aberrantly activated. In sum, RIP3-activated MLKL is further restrained at least at two levels: one through phosphorylation of MLKL on Ser82/83; the other one via protein level regulation, which would fascinate fruitful studies in the near future.

## Supplementary information

Supplementary information, Data and Figures
