# Supplementary material for: Spontaneous necroptosis and autoinflammation are blocked by an inhibitory phosphorylation on MLKL during neonatal development
Source: Cell Res. 2021 Nov 2;32(4):407–10. doi: 10.1038/s41422-021-00583-w (PMC8975819; doi:10.1038/s41422-021-00583-w)

Supplementary information, Data S1

Materials and Methods

Compounds, antibodies and reagents

Recombinant TNF was purified in our laboratory. The Smac mimetic compound was a gift from Dr. Xiaodong Wang's lab which was synthesized as described previously1. Necrostain-1, Dimerizer, and z-VAD are were purchased from Sigma (cat# N9037), Clontech (cat# AP20187) and Bachem (cat# 4027403), respectively. The following antibodies were used in this study: anti-Flag (Sigma, cat# F3165), anti-Myc (Sigma, cat# SAB4700448), anti-pS358-MLKL(human) (Abcam, cat# ab19791), anti-pS345-MLKL(mouse)(Abcam, cat# ab196436), anti-pS223-RIP3(mouse)(Abcam, cat# ab195117), anti-FKBP12 (Abcam, cat# ab108420), anti-hMLKL(Abcam, cat# ab184718), anti-mMLKL (Abcepta, cat# AP14272b), anti-RIP3(mouse)(ProSci, cat# 2283), anti-Flotillin-1 (Cell Signaling Technology, cat# 3253S), anti-CD45 (BD Pharmingen, cat# 561868), Anti-Actin (MBL, cat# PM053-7) , anti-GAPDH (MBL, cat# M171-3).

Plasmids and molecular cloning

The wild-type and mutant forms of MLKL were cloned into the modified lentiviral vector pCDH-CMV-MCS-EF1-copGFP (Addgene) with 3xFlag tag or tandem dimerization domains fusing with Myc-tag (Myc-2FKBPv) at the C-terminus; RIP3 was cloned into the pCDNA3-Myc (Addgene); The wild-type or mutant forms of MLKL N-terminus MLKL(1~178 aa) were cloned into pGEX-6p-1 (GE Healthcare) for expression in E.coli. All plasmids were verified by DNA sequencing.

Cell culture and stable cell lines

HEK293T (human embryonic kidney) cells, HeLa (human cervical cancer) cells, HT-29 (human colon cancer) cells, NIH/3T3 (mouse embryo fibroblast) cells, and L929

(mouse fibroblast) cells were obtained from cell bank of CAS (Shanghai). HeLa-RIP3 cells (for MS analysis) were established as previously described[2]. MLKL deficient cells were generated by using CRISPR/Cas9-genome editing strategy and confirmed by PCR and immunoblotting. The gene knockout strategy has been described in previous study[3] .The sgRNA sequences are as follows: human MLKL (5'-TACTCTTCAAGGACGTGAACAGG-3'; 5'-TTCCCTTAGCAGAATCCACGGGG-3'), mouse MLKL (5'-GCTGAAAGAGATGCATAGAC-3'; 5'-ATGAGAAGCTGAGAGACGTC-3'). Then the HeLa, *MLKL*-KO HeLa or *Mlkl*-KO NIH/3T3 cells were transfected with Myc-tagged human and mouse RIP3 respectively, and the HeLa-RIP3(N-myc), HeLa-RIP3 *MLKL*-KO and NIH/3T3-RIP3 *Mlkl*-KO cell lines were selected with 1 mg/ml G418. These MLKL deficient cells were then infected with lentiviral virus encoding 3xFlag or Myc-2FKBPv tagged wild-type (WT) MLKL or mutated MLKL for further analysis, and the MLKL-expressing cell lines were selected by flow-cytometry. All cell lines were maintained in DMEM (Life Technology, cat# 10569010) supplemented with 10% FBS(Gemini, cat# 900-108), 100 units/ml penicillin/streptomycin (Life Technology, cat# 15140122), and grown at 37°C in a 5% CO_2 incubator.

Cell Survival Assay

About 5000 cells/well were seeded into 96-well plate and allowed to grow for 24 hours. The cells are then treated as indicated. Cell survival was determined using the CellTiter-Glo (CTG) Luminescent Cell Viability Assay kit (Promega, cat# 7573), and the luminescence was recorded with a multimode plate reader (PerkinElmer, Enspire).

Immunoblotting and Immunoprecipitation

Cell pellets were collected and re-suspended in lysis buffer [25mM HEPEs-NaOH, pH 7.4, 150 mM NaCl, 10% glycerol, 1.5% Triton X-100(sigma, cat# T8787)], with complete protease inhibitor (Roche) on ice for 30min. Mouse tissues were

homogenized in buffer A [25 mM HEPEs-NaOH, pH 7.4, 150 mM NaCl, complete protease inhibitor (Roche, cat# 11836145001)] and followed by adding 2% Triton X-100 on ice for 30min. Then the whole cell lysates or tissue lysates were centrifuged at 15,000xg for 30 min. The supernatants were collected for immunoblotting analysis or immunoprecipitation.

For MLKL immunoprecipitation, the lysates of HeLa-RIP3 MLKL expression cells were split into two aliquots, one was incubated with anti-Flag M2 affinity gel at 4°C for overnight, the other aliquot was added with SDS loading buffer and processed as input samples. The next day, beads were washed with lysis buffer for three times and then added with loading buffer as IP samples. Images of western blotting membranes were captured by chemiluminescent gel imager (GE, AI680UV)

Triton X-114 Phase Separation

The aqueous (Aq) and detergent (Det) fractions were separated as previously described[4]. In short, cells were collected and then incubated with 5 x volume of lysis-114 buffer (25mM HEPES, pH 7.4, 150mM NaCl, 2% Triton X-114, and complete protease inhibitor [Roche]). After on ice incubation for 30 min, the cell lysate was centrifuged at 15,000xg for at 4°C for 10 min. Then the supernatant was collected for water bath at 30°C for 5 min, and then centrifuged at 1000xg at room temperature for 3 min. After centrifugation, the turbid detergent fraction (Det) was separated from the aqueous fraction (Aq), which were then analyzed by immunoblotting in reducing or non-reducing conditions with indicated antibodies.

Preparation of Liposomes

Natural and synthetic lipid products (Avanti Polar Lipids) were prepared as 20mM solution in chloroform. The lipid films (2 µmol) of POPC and POPE (with molar ratio 4:1) with or without PI(4,5)P2 and PS (with final concentration 15% and 10%,

respectively) were obtained by evaporating the solvent in the fume hood at room temperature. The Tb^{3+} encapsulated liposomes were prepared by extrusion 24 times through a 100-nm polycarbonate membrane, then washed twice by centrifugation in a TLA-55 rotor (Beckman) at 4°C, which has been detailed described in previous study[4]. The freshly made liposomes were stored at 4°C and must be used within 24 hours.

Liposome Leakage Assay

For the time course liposome leakage assay, aliquots of Tb^{3+} encapsulated liposome with 0.06 μmol total lipid concentration were added with 0.3 ug of wild-type (WT) or mutant MLKL (1~178 aa) recombinant proteins. The excitation (270 nm) and emission wavelength (490 nm) were used for Tb^{3+}/DPA chelates[5], and the fluorescence was continuously recorded with a multimode plate reader (PerkinElmer, Enspire), which was detailed in the previous study[4].

Mass Spectrometric Analysis

RIP3-HeLa cells were treated with doxycycline to induce RIP3 overexpression. Necroptosis was induced by administrating cells with T/S/Z for 16 hours. MLKL bands from RIP3-precipitates were excised in the SDS-PAGE gel. The general steps of MS analysis have been described in previous study[2]. MLKL peptides from necroptotic cells and untreated cells were compared by SILAC quantification, using open-source software MSQuant (http://msquant. sourceforge.net) to calculate the ratios of the heavy/light peptide pairs according to their extracted ion chromatograms (XICs). The quantification results from all peptide pairs were manually checked to ensure their accuracy.

Mouse Strain

All mouse strains were generated in a C57BL/6 background. The *Mlkl* mutant mouse

strains were generated by CRISPR/Cas9-mediated DNA homologous recombination technology and genotyped by PCR and sequencing. Mice were used at the age of as indicated in individual figure legend. All animal experiments were approved by the Institutional Animal Care and Use Committee (IACUC) of Shanghai Institute of Biochemistry and Cell Biology, Chinese Academy of Sciences and complied with all relevant ethical regulations.

Generation of MDFs and BMDMs

MDF generation: Mouse skins were isolated and washed with DPBS for three times. The skins were cut into pieces with tissue scissors and digested with 5mg/ml collagenase I (Life science, cat# 17100017) at 37℃ for 2 h. The digestion was terminated by adding 10% FBS/DMEM medium. After filtered with 45 μM cell strainer, cells were collected by centrifugation at 900 x g for 3 min and cultured in DMEM medium with 10% FBS.

BMDMs generation: Bone marrow-derived cells were cultured in RPMI 1640 Medium (Life, cat# 31800022), which supplemented with 10% FBS and 20 ng/mL recombinant mouse M-CSF (R&D, cat# 416-ML-050). After cultured for 7 days, the attached cells were digested and plated for cell death analysis.

Histology

Freshly isolated mouse organs were fixed in 4% paraformaldehyde for 24 h. The fixed tissues were then dehydrated with ethanol, embedded in paraffin, and sectioned at 5 μm by slicing machine (Thermo Fisher). Tissue sections were analyzed by H&E or IHC staining. For IHC staining, the endogenous hydrogen peroxide was removed with 3% H_2O_2, and antigen retrieval was performed in 10 mM citric acid buffer (pH 6.0) with 0.05% Triton X-100. Mouse tissue sections were then blocked with 2% normal goat serum and incubated with primary antibodies at 4°C for overnight, and then

washed with TBST buffer and incubated with HRP-conjugated secondary antibodies at room temperature for 1 h. After washing for three times with TBST, the signal was developed with 1mmPACT DAB Peroxidase Substrate Kit (Vector, cat# SK-4105). The sections were then counterstained with hematoxylin, dehydrated with ethanol and xylene, and mounted with mounting medium. Images were acquired VS120 (Olympus). For IHC analysis, equal area pictures(512×512 pixels) of each sample were randomly cropped from the overall images (×100) and measured by Image-Pro-Plus.

Cerulein-induced Pancreatitis

Cerulein-induced acute pancreatitis in younger mice has been reported to depend on RIP3-MLKL activation [6-9]. Male *Mlkl*$^{S82E/S82E}$ and their wide-type littermates at 6-8 weeks of age were injected intraperitoneally with 50 μg/kg cerulein (MCE, cat# HY-A0190) or saline every hour for 6 consecutive hours. Two hours after the last injection, mice were sacrificed and the pancreas of each mouse were subjected to histological analysis by H&E staining. Pancreatic necrotic injury was analyzed by counting necrotic pancreas acinar cells, which were determined by morphometry. Five randomly chosen microscopic fields (x 100) were examined for each mouse and the extent of acinar-cell injury/necrosis was expressed as a percent of total image area (512 ×512 pixels). The criteria for acinar-cell necrosis were the presence of acinar-cell ghosts or vacuolization and swelling of acinar cells.

qRT-PCR

Total RNA was extracted by TRIzol Reagent (Thermo, cat# 15596–018), and reverse transcription was performed using the GoScript Reverse Transcription Mix (Promega, cat# A5790). The quantitative PCR analysis was performed with GoTaq qPCR Master Mix (Promega, cat# A6002) with the CFX96 real-time PCR detection system (Bio-

Rad). Results were normalized to the Gapdh mRNA level. Primers sequences used in this study are listed as follows:

mouse Mlkl (forward: 5'-AATTGTACTCTGGGAAATTGCCA-3'; reverse: 5'-TCTCCAAGATTCCGTCCACAG-3'),

mouse TNF (forward: 5'AGTGACAAGCCTGTAGCCC-3'; reverse: 5'-GAGGTTGACTTTCTCCTGGTAT-3'),

mouse Gapdh (forward: 5'-AGGTCGGTGTGAACGGATTTG-3'; reverse:5'-GGGGTCGTTGATGGCAACA-3').

Statistical analysis

All values are calculated from at least three independent biological replicates, unless specifically stated. *p* values of Kaplan-Meier survival curve were determined by two-sided log-rank test. In other tests , the two-tailed unpaired t-test was used. Statistical analysis was performed using GraphPad Prism 8.

References

1 Li L, Thomas RM, Suzuki H, De Brabander JK, Wang X, Harran PG. A small molecule Smac mimic potentiates TRAIL- and TNFalpha-mediated cell death. *Science* 2004; **305**:1471-1474.

2 Sun L, Wang H, Wang Z *et al.* Mixed lineage kinase domain-like protein mediates necrosis signaling downstream of RIP3 kinase. *Cell* 2012; **148**:213-227.

3 Zhang J, Yang Y, Zhou S *et al.* Membrane-bound TNF mediates microtubule-targeting chemotherapeutics-induced cancer cytolysis via juxtacrine inter-cancer-cell death signaling. *Cell Death Differ* 2020; **27**:1569-1587.

4 Wang H, Sun L, Su L *et al.* Mixed lineage kinase domain-like protein MLKL causes necrotic membrane disruption upon phosphorylation by RIP3. *Mol Cell* 2014; **54**:133-146.

5 Wilschut J, Papahadjopoulos D. Ca2+-induced fusion of phospholipid vesicles monitored by mixing of aqueous contents. *Nature* 1979; **281**:690-692.

6 He S, Wang L, Miao L *et al.* Receptor interacting protein kinase-3 determines cellular necrotic response to TNF-alpha. *Cell* 2009; **137**:1100-1111.

7 Wu J, Huang Z, Ren J *et al.* Mlkl knockout mice demonstrate the indispensable role of Mlkl in necroptosis. *Cell Res* 2013; **23**:994-1006.

8 Zhao Q, Yu X, Zhang H *et al.* RIPK3 Mediates Necroptosis during Embryonic Development and Postnatal Inflammation in Fadd-Deficient Mice. *Cell Rep* 2017; **19**:798-808.

9 Newton K, Dugger DL, Maltzman A *et al.* RIPK3 deficiency or catalytically inactive RIPK1 provides greater benefit than MLKL deficiency in mouse models of inflammation and tissue injury. *Cell Death Differ* 2016; **23**:1565-1576.

Supplementary figure 1 legends

(a) Necroptosis analysis of phospho-mimic human MLKL-expressing HeLa-RIP3 cells. *MLKL*-knockout RIP3-expressing HeLa cells (HeLa-RIP3 *MLKL*-KO) were transfected with lentiviral vectors encoding Flag-tagged wild-type or phospho-mimic MLKL (S83D; S83E) as indicated. Apoptosis or necroptosis was induced by administrating cells with T/S or T/S/Z, respectively for 9 hours. Cell viability was evaluated by measuring intracellular ATP levels using CellTiter-Glo assay as described in the Experimental Procedures (lower panel). The data are represented as the mean ± SD of duplicate wells. The expression of MLKL mutants were analyzed by immunoblotting (upper panel). If not stated specifically in this study, 20 ng/ml TNF combined with 100 nM Smac mimetic (T/S) were used to induce apoptosis; 20 ng/ml TNF, 100 nM Smac mimetic, and 20 μM z-VAD (T/S/Z) were used to induce necroptosis.

(b and c) Necroptosis analysis of HeLa-RIP3(N-myc) cells with transient-expression of wild-type and MLKL mutants. HeLa-RIP3(N-myc) cells were transfected with plasmids containing wild-type or MLKL mutants on S83. The cells were selected by flow-cytometry and seeded in 96-well. Necroptosis was induced by treatment of T/Z for indicated time (b) or 6 hours (c). Cell viability was evaluated by measuring intracellular ATP levels using CellTiter-Glo assay (right panel). The data are represented as the mean ± SD of duplicate wells. The expression of MLKL mutants were analyzed by immunoblotting (left panel).

(d) Necroptosis analysis of mutant mouse MLKL-expressing NIH/3T3-RIP3 cells. *Mlkl*-knockout NIH/3T3-RIP3 cells (NIH/3T3-RIP3 *Mlkl*-KO) were transfected with lentiviral vectors encoding Flag-tagged wild-type or phospho-mimic (S82D; S82E) MLKL as indicated. Necroptosis was induced by administrating cells with T/S/Z for 16 hours . Necrostatin-1 (Nec-1, 10 μM), the RIP1 inhibitor, was used to block necroptosis. Cell viability was evaluated by measuring intracellular ATP levels using CellTiter-Glo assay (lower panel). The data are represented as the mean ± SD of duplicate wells. The expression of MLKL mutants were analyzed by immunoblotting (upper panel).

Supplementary Figure 1

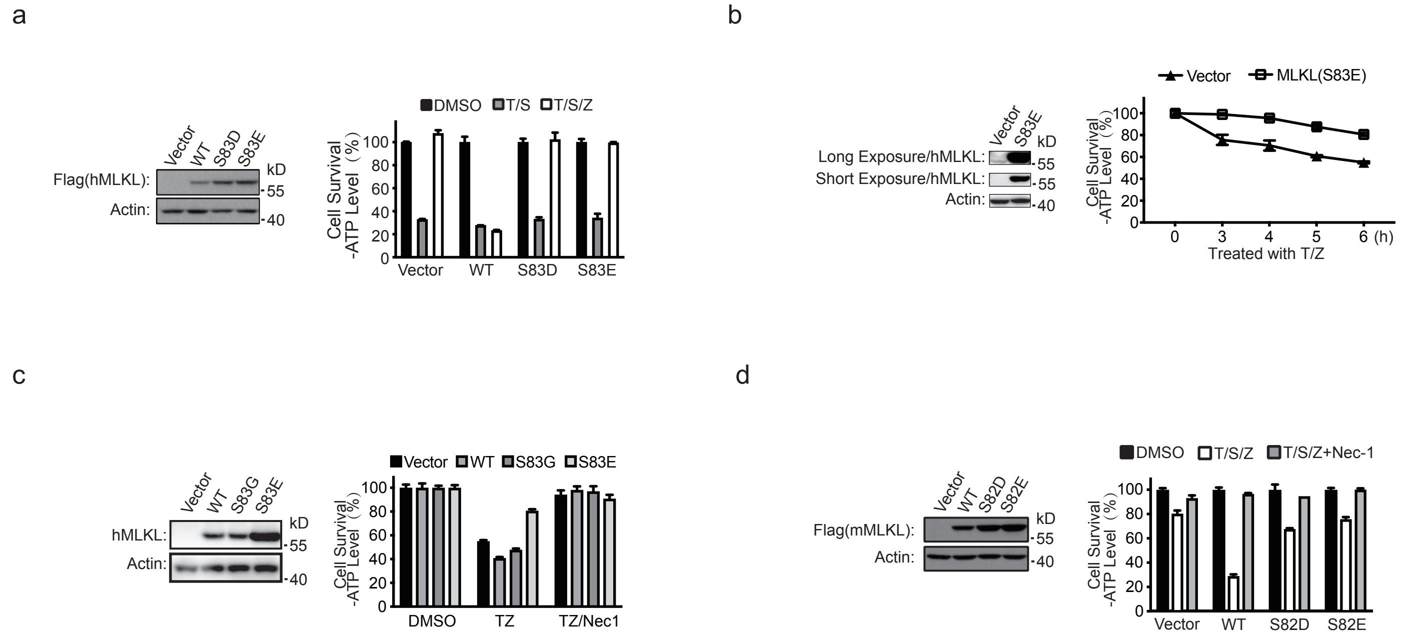

Supplementary figure 2 legends

(a) Schematic strategy of generating *Mlkl*$^{S82G/S82G}$ and *Mlkl*$^{S82E/S82E}$ mice using the CRISPR/Cas9 system.

(b) Representative images of wild-type (*Mlkl*$^{WT/WT}$) and developmentally abnormal *Mlkl*$^{S82G/S82G}$ mice at P8.

(c) Representative images of IHC staining with anti-phospho-mRIP3-S223 antibody on liver sections from *Mlkl*$^{WT/WT}$ and developmentally abnormal *Mlkl*$^{S82G/S82G}$ mice at P8. Scale bar, 50 μm. 3 different views were counted for each mouse. Corresponding quantifications of activated RIP3(phospho-mRIP3-S223 antibody labeled) signals are shown in the right. Each dot represents the data from an individual mouse (n = 3, for each group).

(d) *Tnf* mRNA levels in Liver (left panel) and lung (right panel) were analyzed by qRT-PCR. Organs were obtained from two individual wild-type (*Mlkl*$^{WT/WT)}$ mice, two individual developmental normal S82G (*Mlkl*$^{S82G/S82G}$) mice and two individual developmental abnormal S82G (*Mlkl*$^{S82G/S82G}$) mic at postnatal day 8（P8）. Each dot represents a sample from an individual mouse. Data were normalized to mouse *Gapdh* gene expression.

(e) Representative images of IHC staining with anti-phospho-mMLKL-S345 antibody on a liver section from a developmentally abnormal *Mlkl*$^{S82G/S82G}$ mouse at P8. Scale bar, 100 μm. Five different views were shown in higher resolution (close-up view).

(f) Expression levels of MLKL in liver, lung, and spleen were analyzed by immunoblotting. Organs were obtained from three individual S82G (*Mlkl*$^{S82G/S82G}$) mice and three individual wild-type (*Mlkl*$^{WT/WT)}$ littermates at 8-week-old.

(g) *Mlkl* mRNA levels in Liver, lung, and spleen of mature mice were analyzed by qRT-PCR. Organs were obtained from two to five individual S82G (*Mlkl*$^{S82G/S82G}$) mice and two to five individual wild-type (*Mlkl*$^{WT/WT)}$ littermates at 8-week-old. Each dot represents a sample from an individual mouse. Data were normalized to mouse *Gapdh* gene expression.

(h) Representative images of H&E staining of liver sections from *Mlkl*$^{\mathrm{WT/WT}}$, two developmentally normal *Mlkl*$^{\mathrm{S82G/S82G}}$ mice and developmentally abnormal *Mlkl*$^{\mathrm{S82G/S82G}}$ mice at P8. Scale bar, 20 µm.

(i) Representative image of 10-week-old wild-type (*Mlkl*$^{WT/WT}$) and S82E mutant (*Mlkl*$^{S82E/S82E}$) mice.

(j) Expected and observed numbers of mice per genotype obtained from the intercrosses of *Mlkl*$^{\mathrm{WT/S82E}}$.

(k) Expression levels of MLKL in spleen were analyzed by immunoblotting. Organs were obtained from three individual S82E (*Mlkl*$^{\mathrm{S82E/S82E}}$) mice and three individual wild-type (*Mlkl*$^{\mathrm{WT/WT)}}$ littermates at 8-week-old.

(l) Necroptosis analysis of bone marrow-derived macrophages (BMDMs) isolated from *Mlkl*$^{\mathrm{WT/WT}}$ and *Mlkl*$^{\mathrm{S82E/S82E}}$ mice. BMDMs were isolated from the bone marrow of 8-week-old *Mlkl*$^{\mathrm{WT/WT}}$ and *Mlkl*$^{\mathrm{S82E/S82E}}$ littermates. Necroptosis was induced by administrating cells with T/S/Z for 12 hours. The expression of MLKL was analyzed by immunoblotting (upper panel). Cell viability was evaluated by measuring intracellular ATP levels using CellTiter-Glo assay as described in the Experimental Procedures (lower panel).

(m) Necroptosis analysis of mouse dermal fibroblasts (MDFs) isolated from two individual *Mlkl*$^{\mathrm{WT/WT}}$ and two *Mlkl*$^{\mathrm{S82E/S82E}}$ mice. MDFs were isolated from *Mlkl*$^{WT/WT}$ or *Mlkl*$^{S82E/S82E}$ pups and immortalized. Necroptosis was induced by administrating cells with T/S/Z for 24 hours. The expression of MLKL was analyzed by immunoblotting (upper panel). Cell viability was evaluated by measuring intracellular ATP levels using CellTiter-Glo assay as described in the Experimental Procedures (bottom panel).

Supplementary Figure 2

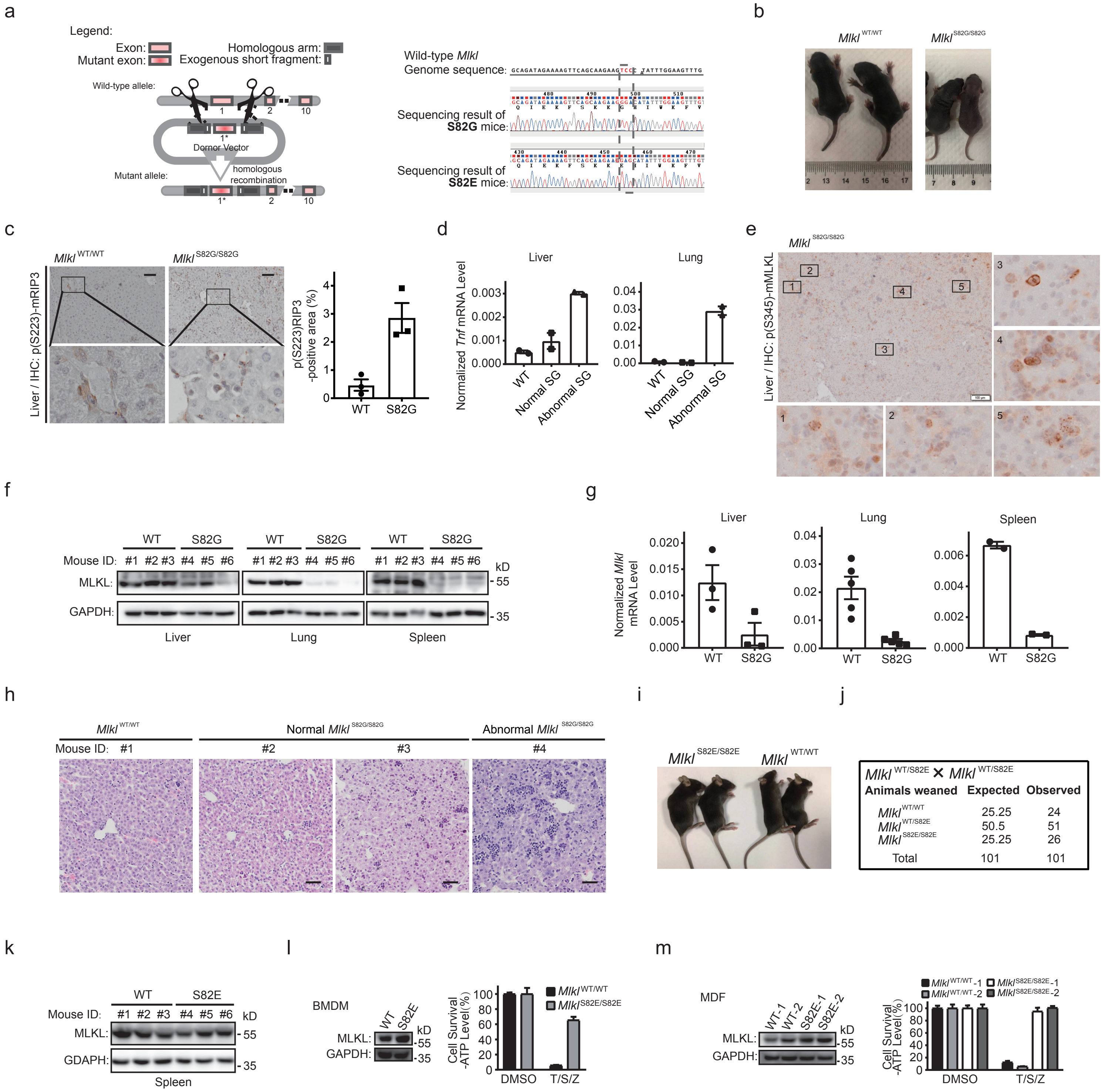

Supplementary figure 3 legends

(a) Time-course analysis of necroptotic cell death in wild-type and phospho-mimic (S83E) MLKL-expressing cells. *MLKL*-knockout HeLa-RIP3 cells were infected with lentiviral vectors encoding wild-type or S83E mutant MLKL as indicated. Necroptosis was induced by administrating cells with T/S/Z for indicated time. Cell viability was evaluated by measuring intracellular ATP levels using CellTiter-Glo assay. The data are represented as the mean ± SD of duplicate wells.

(b) Immunoprecipitation analysis of RIP3 with wild-type or S83E mutant-MLKL interaction upon necroptosis induction. *MLKL*-knockout HeLa-RIP3 cells were transfected with lentiviral vectors encoding Flag-tagged wild-type or phospho-mimic MLKL (S83E) as indicated. Necroptosis was induced by administrating cells with T/S/Z for 8 hours. Cells were harvested and the whole-cell lysates were subjected to immunoprecipitation with anti-Flag M2 beads. The whole cell lysates (input) and the immunocomplex (IP: Flag) were then analyzed by immunoblotting with the indicated antibodies.

(c) Immunoprecipitation analysis of RIP3 with wild-type, S82G or S82E mutant-MLKL interaction upon necroptosis induction. *MLKL*-knockout NIH/3T3-RIP3 cells were transfected with lentiviral vectors encoding Flag-tagged wild-type MLKL or S82G or S82E mutant as indicated. Cells were treated with TNF alone (T) or T/S/Z for 8 hours then harvested and the whole-cell lysates were subjected to immunoprecipitation with anti-Flag M2 beads. The whole cell lysates (input) and the immunocomplex (IP: Flag) were then analyzed by immunoblotting with the indicated antibodies.

(d and e) Phosphomimic mutation on hMLKL-Ser83(d) or mMLKL-Ser82(e) blocked hMLKL dimerization-induced necroptosis. *MLKL*-knockout HT29 cells (d) or L929 cells (e) stably expressing dimerizable wild-type or S82/83E mutant MLKL-2FKBP$_V$ were exposed to dimeric rapalog (dimerizer, 100 nM AP20187) to trigger MLKL activation and necroptosis. The expression of MLKL was analyzed by immunoblotting (left panel). Cell viability was evaluated by measuring intracellular ATP levels using CellTiter-Glo assay (right panel). The data are represented as the mean ± SD of duplicate wells.

(f) MLKL expression-induced necroptosis was blocked by phosphomimic mutation on S82 of mouse MLKL. HEK293T cells were transiently transfected with plasmids containing wild-type MLKL or MLKL mutants (S82E, S345E or S82ES345E). Then, 36 hours after transfection, the cell viability was evaluated by measuring intracellular ATP levels using CellTiter-Glo assay (lower panel). And the surviving cells were analyzed by immunoblotting with the indicated antibodies (upper panel).

(g) Triton X-114 fractionation analysis of wild-type and phospho-S83 mutant MLKL subcellular distribution upon necroptosis induction. Cells were harvested and solubilized in Triton X-114 lysis buffer. Phase separation was performed as described in Figure 1X and the Experimental Procedures. The none-reducing samples were separated by SDS-PAGE and analyzed by immunoblotting using the anti-pS358-MLKL antibody. The reducing samples were analyzed with antibodies shown in Figure 1X. β-actin and caveolar membrane protein flotillin-1 served as loading controls for cytosolic protein and membrane-integral protein, respectively.

(h) Liposome leakage analysis of wild-type and phospho-S83 mutant MLKL. Leakage of Tb^{3+} was induced by exposing liposome to wild-type or S83E mutant recombinant MLKL (lower panel). Asterisks indicated the time point when Triton X-100 was added to induce complete release of Tb^{3+} from liposome. Time course of liposome leakage was monitored. Detailed liposome leakage assay was described in the Experimental Procedures. The recombinant proteins of wild-type, MLKL S83G and S83E mutants (1~178 aa) used in this assay were analyzed by SDS-PAGE with Coomassie-blue staining (upper panel).

Supplementary Figure 3

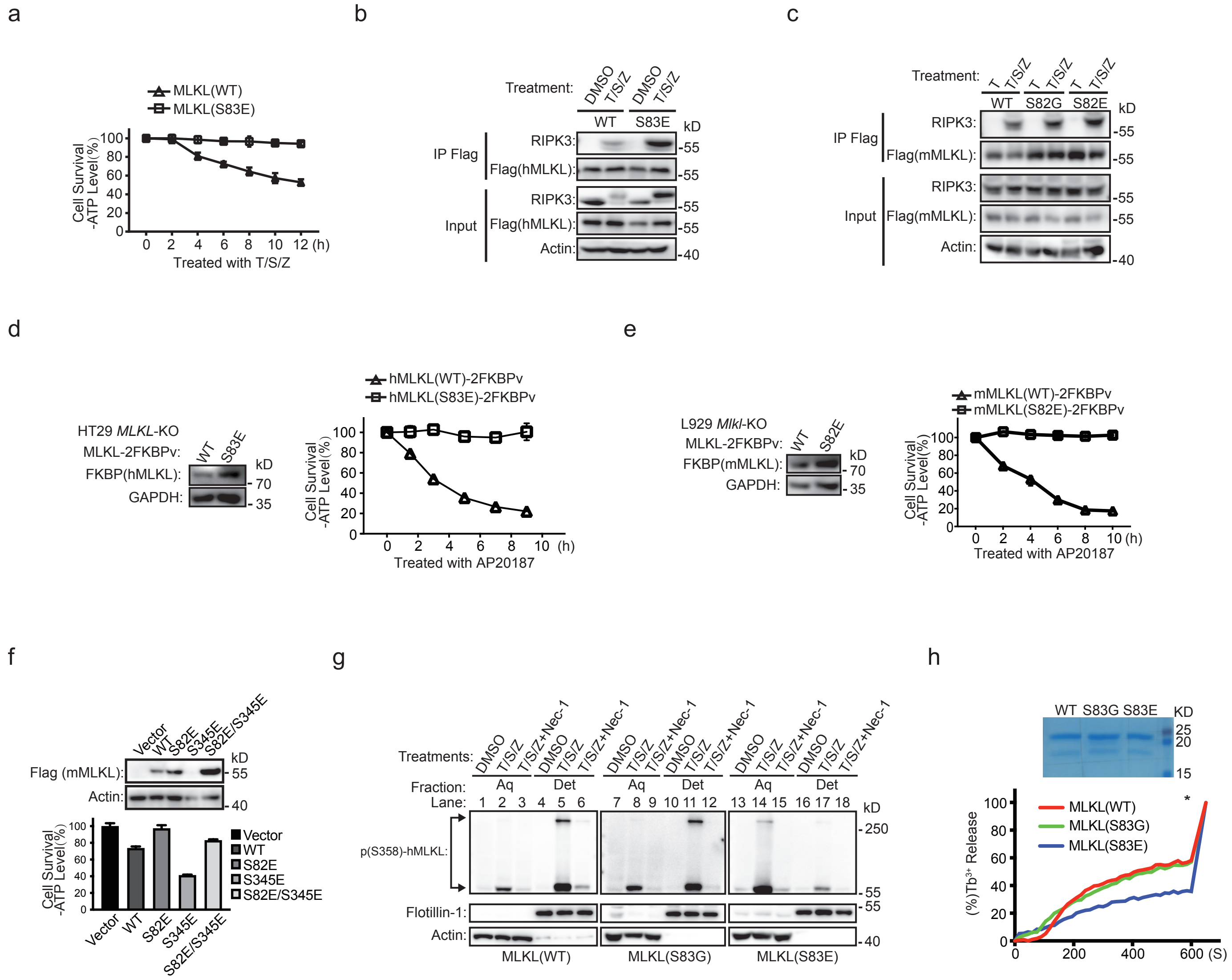

Supplement: Supplementary file 1 — Supplementary information, Data and Figures [file 41422_2021_583_MOESM1_ESM.pdf]
